# Placebo Response is Driven by UCS Revaluation: Evidence, Neurophysiological Consequences and a Quantitative Model

**DOI:** 10.1038/srep28991

**Published:** 2016-07-20

**Authors:** Luca Puviani, Sidita Rama

**Affiliations:** 1 Department of Engineering “Enzo Ferrari”, University of Modena and Reggio Emilia, Via Vivarelli 10, int.1-41125 Modena, Italy; 2 MD at Local Health Unit of Modena, Via S. Giovanni del Cantone 23, 41121 Modena, Italy

## Abstract

Despite growing scientific interest in the placebo effect and increasing understanding of neurobiological mechanisms, theoretical modeling of the placebo response remains poorly developed. The most extensively accepted theories are expectation and conditioning, involving both conscious and unconscious information processing. However, it is not completely understood how these mechanisms can shape the placebo response. We focus here on neural processes which can account for key properties of the response to substance intake. It is shown that placebo response can be conceptualized as a reaction of a distributed neural system within the central nervous system. Such a reaction represents an integrated component of the response to open substance administration (or to substance intake) and is updated through “unconditioned stimulus (UCS) revaluation learning”. The analysis leads to a theorem, which proves the existence of two distinct quantities coded within the brain, these are the *expected* or *prediction outcome* and the *reactive response*. We show that the reactive response is updated automatically by implicit revaluation learning, while the expected outcome can also be modulated through conscious information processing. Conceptualizing the response to substance intake in terms of UCS revaluation learning leads to the theoretical formulation of a potential neuropharmacological treatment for increasing unlimitedly the effectiveness of a given drug.

What is the neural substrate of placebo response? Are there neurons endowed with a special location or specific neurobiological pathways that are necessary or sufficient for placebo response? Recent approaches have attempted to narrow the focus, and a major insight from the recent publications is that there seems not be a single neurobiological or psychobiological mechanism which is able to explain the placebo effect in general. Instead, different mechanisms exist by which placebo (and nocebo) responses originate and exert their actions. Here, we pursue a different approach. Instead of arguing whether a particular neurobiological pathway, mechanisms, or group of neurons contributes to placebo response or not, our strategy is to characterize the kinds of neural processes (at the system-level) that might account for key properties of the more general response to substance intake. With this aim we start analyzing the response to (*open* or *overt*) pharmacological administration. Drug administration is said to be *open* (or *overt*, opposite to *hidden* or *covert*) whenever the administration itself is correctly perceived by a given subject; so that the individual is conscious of the administration act, and, generally speaking, he/she might also be aware of the effects that such a substance will determine on the organism^1^,^2^. We emphasize three properties: pharmacological induced response is *integrated* (the response is given by the integral contribution of different components, which are the *active* pharmacological effect, the *reactive* contribution, due to *self-induced* response, which, in turn is governed by UCS revaluation, conditioning and higher cognitive processing, such as beliefs), at the same time it is highly *differentiated* (one can experience any of a huge number of different pharmacologically-induced response). Furthermore, a third property is represented by the fact that the reactive response mimics the active pharmacological effect (we denote this property as *reactive mimicking*). We first consider neurobiological data indicating that neural processes associated with drug administration response are integrated and differentiated, and, finally, that a distributed system (denoted *reactive system*) mimics the active pharmacological response. We then provide mathematical tools describing the above mentioned properties of the pharmacological response; this leads us to formulate theoretical and operational criteria for determining whether the activity of a group of neurons contributes to placebo response and with which intensity. Moreover, the derived model suggests a pharmacological strategy for increase unlimitedly the response to a drug treatment (and hence the drug effectiveness), exploiting the reactive system dynamics which governs the implicit placebo response.

## Results

### General Properties of (Overt) Drug Administration-Induced Response

Generally speaking, the effect of a drug administration is due to the integration of different processes and components which, together, exert their effect on a given organism.

#### Integration

Integration is a property shared by every response induced by substance intake, irrespective of its specific pharmacological target: Each response comprises a single overall effect that cannot be decomposed into independent components. More specifically, drug administration, and generally speaking substance consumption, exerts an overall effect which is perceived as unique, conveying pharmacological effects, rewarding, expectations, beliefs, desire, emotional feelings, filtering processes (e.g. *somatic focusing attention*) and prior experiences^1^,^3^,^4^. A striking demonstration is given by the decreased effectiveness of hidden treatments. In particular, hidden drug administrations eliminate the psycho-social (placebo) component, so that only the pharmacodynamic effect of the treatment (free of any psychological contamination) can be accomplished. More specifically, in the open versus hidden treatments paradigm^1^,^2^,^5^,^6^ drugs are administered through hidden infusions by machines; such infusions can be administered using computer-controlled infusion pumps that are pre-programmed to deliver drugs at a desired time. The crucial factor is that the patients do not know that the drug is being injected. The difference between the responses following the administration of the overt and covert therapy is the placebo (psychological) component, even though no placebo has been given^1^. Furthermore, experimental results^5^ show how the overall response to an analgesic pharmacological treatment is due to the contribution of 1) prior conditioning and implicit learning, 2) expectations and 3) active pharmacological effect; moreover, the three above mentioned contributions are indirectly evaluated through the involvement of different treatment groups in double-blind design, exploiting the open versus hidden paradigm.

Moreover, recent studies have shown that the treatment (or drug) response, for a given subject, changes if the type of administration varies between open and hidden over time. This effect does not depend on the specific disease, since it can be reproduced, at least, in pain, anxiety, and Parkinson’s disease (PD)^2^,^7^. For instance, in subthalamic nucleus– deep brain stimulation (STN-DBS), PD patients aware of the beginning of the stimulation (open condition) show higher motor improvements than when they are hiddenly stimulated; on the other hand, patients aware of the interruption of the active stimulation (open interruption) show quicker and deeper motor impairments than when an hidden interruption occurs. In these examples it is evident how an adding contribution (which is due to previous interactions with the treatment) add up to the active treatment effect whenever the subject’s brain infers the presence of such a treatment. Provided that in the above mentioned examples subjects have already experienced such treatments in the past, it can be assumed that expectation is negligible with respect to the contribution due to previous learning. On the contrary, whenever an open versus hidden treatments paradigm is applied for a new drug or treatment, the placebo contribution can be a mixed combination of expectation and (similar) previous learned treatments.

#### Differentiation

While the drug administration response is given by an integral contribution of different components, there are experimental evidences which show how the mammalian brain can finely discriminate between administration (and/or the intake) of different substances. For instance, neurons in rats can discriminate between cocaine and liquid rewards^8^,^9^, or between cocaine and heroin^10^, possibly even better than between natural rewards^11^,^12^.

At this point it is useful to introduce some definitions. Provided that a given drug administration represents an *unconditioned stimulus* (UCS), an *active response* is defined as the response determined by the active pharmacological effect of the UCS within the central nervous system (CNS). Moreover, a *reactive response* is defined as any response which is determined by a self-induced reaction within the CNS, without any active pharmacological contribution. For instance, a conditioned response (CR) induced after some pairings of an active pharmacological UCS and a neutral conditioned stimulus (CS) corresponds to a reactive response, since the elicited response is not sustained by any pharmacological active effect. For this reason, a reactive response could represent a specific form of placebo. Furthermore, the UCS *implicit learning* is defined as the implicit (or automatic) UCS response (UCR) evaluation and re-valuation (i.e., UCR *inflation* and *devaluation* or *deflation*; e.g., see refs 13, 14, 15).

#### Difference between (pharmacological) conditioning and implicit UCS revaluation

When a neutral CS is paired with an active drug, the associative learning process is called classical conditioning^16^, (for instance when a tone is paired with the self administration of cocaine in rats^17^); furthermore, when a drug response (UCR) is evaluated and revaluated over trials the learning process is represented by UCS revaluation^14^. In other words, from one hand, classical conditioning learning occurs whenever the CS and the UCS corresponds to different discriminable elements, such that the organism can learn the simultaneously (or causal) co-occurrence of both elements (in other words the “association” of two distinct elements is learned^16^). Instead, on the other hand, in successive trials involving drug administration there is a unique element (i.e. the drug administration itself, which represents the UCS), which determines an UCR which, in turn, could vary during successive administration trials; more specifically, if the dose of a drug (i.e. the UCS active stimulation intensity) changes over successive open administration trials, the organism updates the evaluation of the experienced outcome through UCS revaluation (for instance, in the literature experiments related to UCR revaluation, independently from classical conditioning, are reported^13^,^15^,^18^; furthermore, a quantitative analysis of UCS revaluation and its relation with classical conditioning have been derived^14^). It is worth noting that, generally speaking, both classical conditioning and UCS revaluation could occur during drug administration, since the context and other cues could be associated as CSs with the drug itself (UCS); moreover, such CSs, may help recognizing the given drug in successive administration trials. Furthermore, specific CSs, such as flavors, odours and others, could be implicitly misattributed to be (at least partially) the causal element of the drug effects (UCR). Finally, we will show in the following Sections how implicit UCS revaluation drives classical conditioning (and, generally speaking, inferential learning), so that specific pharmacodinamical curve can be learned/mimicked by the CNS.

Generally speaking, a response induced within the CNS can be represented by the superposition of the activity of different neural populations; more specifically, assuming that the CNS consists of *N* different neuronal populations, the response, denoted with the vector **y**, can be expressed as:


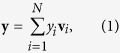


where *y*_*i*_ represents the i-th neuronal population activity and {**v**_*i*_; *i* = 1, 2, …, *N*} represents a set of versors, being associated with different neuronal populations, which form a complete basis 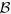 for the CNS space. In particular, *y*_*i*_ is a real quantity representing the product between the mean number of elicited neurons and their mean firing rates for the *i*-th neuronal population (with *i* = 1, 2, …, *N*); consequently, *y*_*i*_ takes on a positive (negative) value if the response produces an increase of (a decrease or inhibition of) the activity for the *i*-th population, and is equal to zero whenever the response does not involve any adjustment for the baseline activity of the population. It is worth noting that the different neuronal populations could be interdependent (i.e., 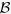 does not represent an orthonormal basis), and, the CNS is a nonlinear system, so that even small variations of the activity (or the elicitation) of a single response component could lead to a very different outcomes (for instance, a small variation of the mean firing rate of a population, or of the quantity of a given neurotransmitter, can even lead to an opposite behavioral outcome^19^).

#### Reactive mimicking

In a growing body of literature it has been reported that pharmacological conditioning (and implicit UCS revaluation) determines reactive responses which mimic the original active pharmacological effects. For instance, experimental results^17^ show that an increase in dopamine release in the *ventral striatum*, measured through microdialysis, are observed not only when rats self administer cocaine (UCS), but also when they are solely presented with a tone (CS) that has been previously paired with cocaine administration. Furthermore, it is known that pharmacological conditioning, like conditioning with opioids, produces placebo analgesia mediated via opioid receptors, and that, if conditioning is performed with non-opioid drugs, other non-opioid mechanisms are involved, so that conditioning activates the same neuronal populations as the active pharmacological treatment^5^. Similar results and conclusions have been obtained in mice experiments^20^. Moreover, brain imaging data, evidence that placebos can mimic the effect of active drugs and activate the same brain areas; this occurs for placebo-dopamine in Parkinson’s disease^21^,^22^, for placebo-analgesics^5^,^20^,^23^,^24^,^25^,^26^ or antidepressants, and for placebo-caffeine in healthy subjects^27^. It is worth noting that since the active pharmacological response is highly differentiate, the reactive mimicking property leads to the highly differentiation of the *reactive system*. Such a system can be thought as a distributed network within the CNS, which generates reactions (or self-made responses) to relevant stimuli and substances.

The above mentioned properties of pharmacological responding, that are, integration, differentiation and reactive mimicking, can be described from a quantitative perspective; this should lead to the derivation of the laws governing the origin and the dynamics of the placebo response. To this aim it is also necessary describing the dynamics of the UCS revaluation, that is the implicit UCR evaluation over successive administration trials.

It is worth noting that the above mentioned properties (and the analysis which follows in the next sections) hold for either the placebo and nocebo response. Indeed, a “negative response” (nocebo) can be easily described as an increase of the activity of a given neuronal population (e.g. anterior insula) and/or as an inhibition of the baseline mean firing rate of the target population (e.g. inhibition of dopamine neurons activity within a specific brain region; see Eq. (1)), exactly like a positive (placebo) response.

### Error-Driven Learning

From a growing body of literature emerges that learning occurs through the computation of specific *error-signals* (or *prediction errors*)^28^,^29^. Generally speaking, the prediction error is defined as the difference between the response expected from a given stimulation and the response actually perceived by the elicited organism. This definition relies on experimental observations acquired in functional imaging studies^28^,^30^,^31^, or directly measured in dopaminergic circuits (e.g., in the *ventral tegmental area*, VTA) or in other fear-related circuits^12^,^29^,^32^,^33^,^34^,^35^,^36^,^37^,^38^.

Different mathematical models describing classical conditioning learning (e.g., Rescorla-Wagner model^39^,^40^ or *temporal difference* (TD) *models*^31^,^41^,^42^,^43^), or describing learning in general, such as the probabilistic (Bayesian) “perception” and “action” learning models (i.e., the *predictive coding* (PC)^44^,^45^ and the *active inference*^46^,^47^), assume that coding behavioral responses involves the computation of an error-signal. More specifically, the brain makes predictions in relation to a given stimulus and, on the basis of the experienced outcome, the prediction is updated through the prediction error. If the experienced outcome is greater (lower) than the prediction, the computed error signal is positive (negative) and corrects the new prediction; furthermore, if the experienced response coincides with the expected outcome, the error signal is zero and no prediction updatings take place.

### Theoretical Concepts and Quantities

#### Differentiation

Eq. (1) describes the elicited response within the CNS accounting for all the involved neuronal populations (or response components); however, we can consider one single component to ease the reading. This choice, however, does not entail any loss of generality, since our model can be applied to any component.

#### Integration

As previously shown, the response to a drug administration can be expressed by three main contributions (each of which might be further decomposed in more detail), these are: 1) the active pharmacological response, denoted *x*; 2) the reactive (self-induced) response due to implicit UCS revaluation learning and classical conditioning, denoted *i*_*R*_; 3) the reactive response due to expectations and, generally speaking, to higher cognitive information processing (such as beliefs, social observations, desire, wanting, attentional mechanisms and verbal suggestions), denoted *γ*. A fundamental question is “how all these contributions can be integrated within the target neuronal (network) population (i.e. the target component)”? To answer this question it has to consider that, even if a single neuron presents a chaotic and non-linear dynamics, an overall neural network presents a deterministic and linear input-output transfer function^48^,^49^. More specifically, recent computational and *in vivo* analysis have evidenced that cortical circuit have recurrent excitatory and inhibitory connections^48^,^49^,^50^,^51^,^52^. Such a network architecture comprises excitatory and inhibitory neuronal populations, and the connectivity could be random and sparse. Computational studies about large networks reveal that the dynamics tends to a natural stationary state called *balanced state*. In this state, a balance between the excitatory and inhibitory inputs emerges dynamically for a wide range of parameters, and the internal synaptic inputs act as a strong negative feedback, which linearizes the population responses to the external drive despite the strong non-linearity of the individual cells. For these reasons, at a neural network level, the linearity hypothesis holds, and different contributions (in particular either excitatory or inhibitory contributions which determine an increase or a decrease of the mean target population firing rates) add up linearly (*superposition principle*). Hence, the integration property leads to the following expression for the general elicited neuronal population:


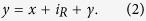


It is important to note that the above described “response decomposition” is only a theoretical abstraction, since, because the integration property, the response is unique and the brain cannot discriminate between the different contributions. In other words, considering a given neuronal population (component) the brain cannot discriminate between the contributions due to desire, expectations, implicit processing and active pharmacological effects.

#### Reactive mimicking

Since the reactive response (*i*_*R*_) associated with a given substance determined by implicit UCS revaluation or by pharmacological conditioning mimics the active pharmacological effect (*x*), the reactive response for a given neuronal population can be expressed as:


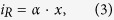


where the term *α* takes on real values and represents the *efficiency* (or the *strength*) of the reactive system for the given neuronal component.

#### Implicit UCS revaluation

If multiple successive open drug administration trials are considered, provided that no conscious information are available (such as verbal suggestions or beliefs) about the drug effects, it can be assumed that in every trial the expected (or predicted) response coincides with the last experienced outcome (which, in turn, coincides with the response experienced in the previous trial, recursively), or, alternatively, that the expected response converges over successive trials to the actual experienced outcome. Without any loss of the generality, we can assume that the predicted outcome is equal to the last experienced outcome. Provided that the active pharmacological effect (*x*) is constant over successive drug administration trials, and that the higher cognitive information processing are avoided (in other words, *γ* = 0, see Eq. (2)), the reactive response due to implicit learning (i.e., due to the UCR, or *y* evaluation and revaluation over trials), *i*_*R*_, is updated through the error-signal computation. In the first trial the reactive response is equal to zero, since no previous learning occurred for the given drug administration (UCS); hence, *y*_1_ = *x*. Since the expected outcome was equal to zero for that UCS, the error signal after the first administration trial is equal to *x* and, such a prediction error updates the reactive response, according to Eq. (3). In the second open drug administration trial the response is given by Eq. (2) with *γ* = 0, that is *y*_2_ = *x* + *αx*; moreover, a new error signal is computed as *y*_2_ − *y*_1_ = *αx*, and the reactive response updated at the end of the second trial is given by *i*_*R*2_ = *αx* + *α*^2^*x*. It easy to demonstrate (see Methods) that the response elicited in the n-th drug administration trials can be expressed as:


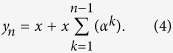


Eq. (4) shows that the response to a given administration drug diverges (to infinity) or converges to an asymptotic value, as the number of trials increases, depending on the magnitude of *α*. The experimental observations (and the ordinary life experiences) show that the pharmacological responding tends to reach an asymptotic value over successive trials and does not diverge; hence the absolute value of *α* has to be comprises between zero and the unity (i.e., 0 < |*α*| < 1). We denote the above mathematical condition as *stability of the reactive system*. Under such a condition it is easy to demonstrate that the asymptotic response can be expressed as follows:


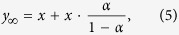


where the active pharmacological effect (*x*) and the reactive component learned during successive trials (i.e., the quantity given by 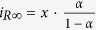), are highlighted. At this stage, if in a successive trial an inerte substance is administered (in other words, if a placebo is given, so that the active pharmacological effect is brought to zero, *x* = 0), the placebo response coincides with the reactive response *i*_*R*∞_, implicitly learned over trials. We argue that such a reactive response represents the *unconscious* or *implicit placebo response*. If the placebo is administered during successive trials, it is easy to demonstrate that the response tends asymptotically to zero, driven by the error signal (see Methods). It is important pointing out that the open administration (UCS) allows the individual to perceive the UCS (i.e., the drug administration itself), and hence to trigger the learned reactive response associated with that UCS; furthermore, an error signal is computed whenever the UCS outcome is revaluated. Conversely, if the administration was hidden, no UCS can be perceived and no reactive response can be triggered. Moreover, it is arguably that different features of the drug administration (or substance intake), such as the flavor of a given pill (or even conditioned stimulus), contribute to perception and differentiation of the given substance intake (UCS).

Finally, the *integration*, the *reactive mimicking* and the *stability of the reactive system* properties lead to the following theorem:

**Theorem 1** *It is necessary that two distinct quantities are encoded within the CNS for the stability of the reactive system, these are the reactive response and the predicted* (*expected*) *outcome*.

The demonstration is given in Methods. In practice, the theorem proves that both the reactive response (*i*_*R*_) and the expected (or predicted) outcome have to be computed and independently updated in order to assure the stability of the reactive system. In other words, the theorem proves that the reactive response cannot be represented by the predicted response, or, equivalently, that expecting a given response does not coincide with the “self-induction” of that response.

### Outcome Simulation and Conscious Placebo/Nocebo Response

Since the above mentioned theorem proves that both the reactive response and the expected outcome have to be coded within the CNS, the response to drug administration (more specifically the reactive component of it) should be a function of these quantities. In the previous Section it is shown how, in the absence of cognitive information processing, the reactive response is implicitly updated by UCS revaluation learning through the computation of the error signal. In this Section it is proposed how it is possible modulating the reactive response through cognitive and conscious information processing, such as verbal suggestions, beliefs, expectations, and even by social observational learning^53^. More specifically, we hypothesize that cognitively processed information, which permits to infer (i.e., *to simulate*) the outcome of a given substance intake, lead to a process that we denoted *outcome simulation*. Such a process consists of high level cognitive processing of different pieces of information related to the outcome, and lead to the computation of the most probable outcome. Certainly such a computation depends also on the previous experiences which are evoked by the provided information. If the simulated outcome is different from the expected outcome (i.e., from the previously experienced outcome) an error signal is computed and the reactive response (*i*_*R*_) is updated accordingly. From a mathematical and modeling perspective, the outcome simulation process corresponds to a *virtual* substance administration trial, in which the experienced outcome is the simulated (predicted or inferred through cognitive processing) outcome, and the error signal is computed as the difference between the simulated and the expected. It easy to prove that the reactive response is updated by the error signal through the following recursive expression^14^:


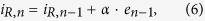


where *e*_*n*_ represents the error signal computed in the (n-1)-th trial (real or virtual), provided that *α* does not change over time; otherwise, the more general expression for *i*_*R*_ in the n-th trial is as follows:


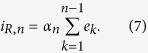


For these reasons, verbal suggestions or beliefs which let a given individual inferring a positive (negative) outcome, lead to the computation of a positive (negative) error signal which increases (decreases) the reactive response associated with a given drug or treatment. We argue that the simulation process takes place not only in drug administration but even in more general situations, such as in stimulus-outcome learning (or UCS evaluation); for instance animals could learn that a given stimulus is dangerous observing others facing with such a stimulus^54^. Moreover, cognitive information processing could influence the reactive response associated with different stimuli and substances; for instance, functional MRI studies have shown how prices of wine bottles (which represent here a piece of information related to the outcome) can affect the experienced pleasantness of the wine intaking (UCS)^55^. Indeed, the experienced pleasantness (which represents here the UCR) is due to the integration of the active component, *x*, and the reactive (self-induced) response *i*_*R*_, which is due to prior learning and/or cognitive evaluation. This line of reasoning is also supported by experimental results^37^,^56^ which show that inferred outcomes (e.g. rewards), never directly experienced before, determine prediction errors computed in the VTA, which are just like errors based upon direct experience/stimulation. In practice, this means that the inference/simulation process results in the computation of error signals on the basis of the difference of the actual expected outcome and the inferred outcome (through information processing) associated with a given UCS or situation, before experiencing a direct active stimulation. Such errors update the reactive response associated with the given UCS (drug or treatment) through Eq. (6), modulating the placebo response.

Concluding, cognitive information processing could lead to an outcome simulation process which, in turn, modulates the reactive response through the computation of an error signal, understood as the difference between the simulated (inferred) outcome and the expected (previously experienced) outcome.

### Placebo response due to *misattribution* processes

It is well known that placebo response can also be induced by emotional feelings, wanting, desire and filtering processing such as attentional somatic focus^4^. Since emotions and all the above mentioned processes produce specific neuronal responses, it is important to note that if such responses are misattributed to a given (even inert) drug or treatment (UCS), an UCS evaluation (revaluation) process occurs, determining the computation of an error signal. In practice, the misattributed response add up directly to the drug response (if exists) so that a prediction error is computed. Whenever an emotional response due to a source of stimulation is attributed to a “ wrong” source, an event of source misattribution occurs^57^,^58^,^59^. It is worth pointing out that misattribution may result either from conscious, accessible and measurable controlled processes, or from spontaneous, inaccesible, automatic processes^60^,^61^ (i.e. implicit misattribution). The placebo response induced through the misattribution of a purely reactive (self induced) response can become a resistant-to-extinction reaction, since, in this particular case, the expected response becomes equal to the elicited reactive response and no error signal is further computed. Such placebo responses can be so well established that they can persist even after the given subject has been informed about the fact that the administered drug is a placebo^62^. This effect cannot occur through reactive mimicking, since as soon as the expected active component drops to zero (a placebo is given) a negative error signal is computed and it leads the response toward zero.

### Equivalent model in continuous time scale - Time course of drug response

The analysis and the model shown in the previous sections hold if the active drug stimulation can be approximated with a constant value in every trial. Furthermore, the time duration of such trials has to be relatively short with respect to the inter trial intervals; in other words, the time duration of a trial can be approximated as a point in the time axis so that a discrete model can be adopted. Despite this scenario has been reproduced in laboratory experiments for the study of placebo^63^,^64^,^65^, real world drugs determine specific pharmacodynamical curve over time (i.e. specific pattern or time course effect which cannot be approximated as a constant value, in other words, a time function *x*(*t*) exists, in place of a constant *x*, see Eq. (2)). For these reasons it is important to derive a continuous time model starting from its discrete counterpart, in order to properly describe the drug response over time (and its active and reactive contributions). The continuous model derivation is proposed in Methods, while in the next Section computational results from such a model are proposed in order to obtain a comparison with experimental measures of hormonal placebo response^66^,^67^. It is worth noting that prediction errors (due to continuous UCS revaluation over time within a given drug administration trial) drives classical conditioning learning, so that the specific pharmacodynamics of a given drug is learned through successive trials. Indeed, time instants from the onset of drug administration can be considered as contextual cues (CSs) and the responses associated with such cues represent the related UCRs (which are shaped by prediction errors). This line of reasoning is supported by experimental measures which show that unconscious placebo mimics the pharmacodynamical effect of a given drug^66^,^67^.

### Operational Measures and Prediction of Implicit Placebo Response

Given a specific target neuronal population, the active response to a pharmacological administration (*x*) could vary across individuals, and, more important, the reactive response component (i.e., the implicit placebo or reactive response, *i*_*R*_) could be even more variable. Indeed, the parameter *α*, which drives the reactive dynamics, should be a function of different parameters, such as the specific neural population involved (such as dopaminergic, opioidergic, serotonergic, and so on) and the number, the types and the sensitivity of the receptors within the given population. In turn, these variables depend on different parameters, for instance these are functions of the individual’s genetic makeup. Indeed, there is growing evidence that the individual’s genetic makeup (a stable trait) influences clinical outcomes and potentially may allow for identification of placebo responders^68^. More specifically, treatment outcomes in the placebo arms of trials that have assessed genetic variation in the dopaminergic, opioid, cannabinoid, and serotonergic pathways suggest that genetic variation in the synthesis, signaling, and metabolism of these neurotransmitters contributes to variation in the placebo response^68^. For these reasons, it could be useful to estimate (i.e., measure) the quantities *x* and *α* for a given pharmacological target and for a specific genetic makeup (exploiting the so-called *personalized medicine*^69^). With such measures it should be possible to optimize the combination *drug-genetic makeup* and considering the implicit placebo response as a stable and reliable contribution of the pharmacological responding and, hence, as an indistinguishable part of the “true” *pharmacological treatment effect*. Operationally, the measure of the active pharmacological response can be assessed by hidden or covert drug administrations. The response should be assessed quantitatively adopting, for instance, functional brain imaging or neurons activity recording, estimating the activity (understood as the product of the mean number of firing neuron and the mean firing rates) increase of the target neuronal population. Furthermore, the parameter *α* can be assessed by open (overt) drug administration, provided that the subject does not know which is the pharmacological target^5^ (in other words the reactive response due to implicit UCS evaluation is considered, and the expectations, beliefs of clinical benefit or higher cognitive information processes are avoided; i.e. *γ* = 0, see Eq. (2)), or for specific outcomes which are not susceptible to expectations, such as hormonal responses^66^,^67^. More specifically, assessing the increase of the given neuronal population activity in successive drug administration trials permits to estimate the increase of the overall response (which comprises the active and the reactive response) which will converge to an asymptote, according to Eq. (5). Moreover, the asymptotic convergence to zero of the activity of the given neuronal population can be assessed over successive placebo administration trials. Hence, the parameter *α* could be reliably estimated from these data through simple mathematical computations. It is worth noting that such measures have to be determined for single individuals and, successively, for groups with uniform target genetic traits. In fact, it is expected that the parameter *α* presents a notable variance across subjects and genetic traits, as emerges also by observing the increase of variability of responses from hidden (determined by *x*) to open (determined by *x* and *α*) analgesic drugs administration^70^. Hence, actual clinical and pre-clinical trials cannot be adopted to extrapolate the *α* parameter for a given target population, since traditional randomized placebo controlled trials (RCTs) are optimized to demonstrate pharmacological efficacy (i.e. the active contribution, *x*) at the group level. While RCTs are ideal for conclusively demonstrating pharmacological efficacy in the “average patient”, they cannot be informative about the time course of the implicit placebo response over successive trials. Moreover, the outcome measure from RCTs are often specific scales (e.g. pain scales, or motor performance scale) and not the direct neuronal population activity. Another important issue is represented by the need to avoid the higher cognitive contributions (e.g. cognitive expectations, beliefs, and information about the drug target, which is represented by the parameter *γ* in Eq. (2)), in order to estimate the active and the reactive contributions. For this reason animal trials can be adopted for the model assessment. However, open versus hidden paradigms within individuals with a trained experience with a given treatment or drug can be adopted for the estimation of the *α* parameter, since in this case the expectation is negligible with respect the learned implicit reactive response. Nevertheless, even in this case, single subjects and single homogeneous genetic traits have to be considered for a proper assessment. The above mentioned issues demonstrate the necessity to design specific trials for the assessment of the implicit placebo response.

**Figure 1:**
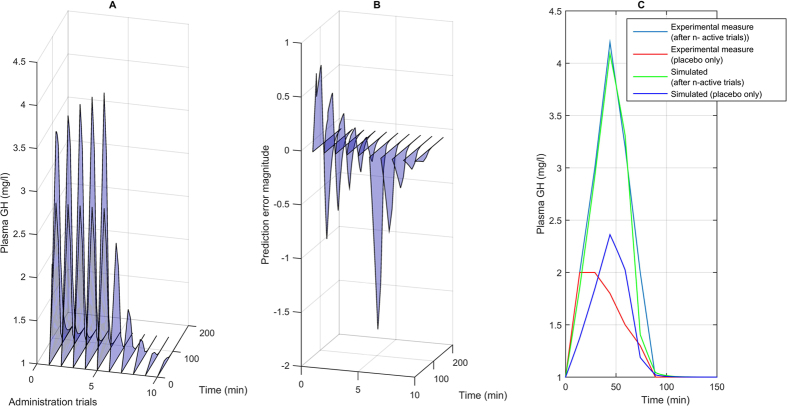
1A: responses (understood as plasma GH concentrations) over time (within each trial) and over successive 5 − *HT*_1*B*/1*D*_ administration trials are simulated. More specifically, in the first 5 trials the drug is actively administered, so that the implicit learning (and reactive mimicking) takes place, after that, starting from the 6-th trial a placebo is administered. In Fig. 1B the time course of the prediction error over successive trials is computed. Finally, in Fig. 1C the experimental measures and the computational results are compared. In particular, four different curves are reported: a) the overall response after *n* trials for two consecutive days (experimental^66^,^67^), b) the overall response after 5 active administration trials (such that the asymptotic response has been reached) from numerical simulations, c) the first placebo response after n active administration trials (experimental measure) and d) the computed placebo response after the 5 active trials. Model parameters adopted for the simulation: *T*_0_ = 150min; *τ* = 10min; *α* = 0.45; 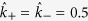; 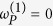; 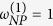. The experimental measured values have been extrapolated from published data^66^,^67^ (for instance see Figure 5D, pag. 4320^66^).

Nevertheless, it has been shown that induced hormonal responses, for instance administering the serotonin agonist sumatriptan (5 − HT_1*B*/1*D*_), determine a placebo response exclusively determined by previous implicit learning and not by expectations or beliefs (i.e. *γ* = 0). Indeed, it has been shown that either positive or negative expectations do not change the placebo response, while previous learning determine a reactive response that mimics the pharmacodynamics of the last administration. Hence, hormonal response could be a good candidate for the evaluation of the model (since only unconscious placebo/reactive mimicking takes place and no expectations or higher level cognitive information processes are involved). If it is assumed that in the above mentioned case, the given target CNS population activity is linearly correlated with the growth hormone (GH) release, the continuous time model can be adopted in the computation of the active and placebo responses over time (within a given trial) and over successive trials. More specifically, Fig. 1 shows a comparison of the experimentally measured plasma concentration of GH over time after n successive pharmacological active trials (developed in two consecutive days), and in the third day with placebo administration^66^,^67^, with the computational results obtained from numerical simulations with the continuous time model. The results show that the model is able to describe the overall drug response (active plus reactive contributions, over time and over successive trials) and the placebo response without active component. Nevertheless, further experimental trials have to be developed in order to validate the model and for parameters fitting, taking into account the issues illustrated above (e.g. the measured response has to be the CNS population activity or, alternatively, a mapping function between such a response and the desired outcome or scale has to be determined previously, homogeneous genetic traits or single individuals have to be considered, expectations and beliefs contributions have to be avoided, and so on).

### Drugs that alter the function of brain’s communication system

Certain drugs are able to induce long-term neurobiological changes; for instance, cocaine alters the dopamine communication system promoting a rapid up-regulation of dopamine transporter (DAT) expression on the cell surface. In particular, one-time use of cocaine will increase the surface DAT expression for at least a month, since the normalization of dopaminergic function is usually an extremely slow process^71^, so that, there are less dopamine molecules available in the synapse for signaling. In this case the baseline activity of the target neuronal population (i.e. dopamine neurons) changes over time and over successive drug administration trials (in particular it decreases, see also the definitions associated with Eq. (1)). Other drugs and treatments (and even some diseases) can alter the neurobiological base activity levels and communication mechanisms, such as antidepressant (which promote neurogenesis), or antipsychotics, which determine a time-dependent reduction in the number of spontaneously active dopamine cells^72^, and others. In all these cases, the actual baseline activity level for the target population has to be assessed before drug administration. In fact, the reactive contribution (i.e. unconscious placebo response) is still determined by the accumulation of the error signals over successive trials (see Eqs (6 and 7)), but it could be completely masked or apparently increased if such a baseline adjustment is not properly accounted for. From a modeling perspective, the overall response in the generic *n*-th administration trial can be expressed as:


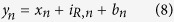


where, the term *b*_*n*_ represents the baseline activity with respect to the baseline level that was present before any open drug administration.

### Obtaining Increasing and Resistant-to-Extinction Reactive Responses

Since the mammalian brain is able to induce reactive responses similar to those obtained by pharmacological active effects, exploiting the implicit reactive system dynamics for obtaining a desired outcome should be feasible, in principle. More specifically, our system-level model suggests that it could be possible obtaining resistant-to-extinction reactive responses manipulating the error signal computation. Indeed, even avoiding and neglecting cognitive processing (e.g., verbal suggestions) during drug administration or substance intake, as previously shown, the error signal is implicitly computed, and it drives (or updates) the reactive response *i*_*R*_ associated with the representation of the given drug (i.e. the UCS; see also ref. 14 for a more comprehensive analysis). Provided that, for a given genetic makeup and for a specific target CNS component, the reactive system efficiency *α* is greater than zero, after some active drug administration trials the reactive response reaches the asymptote 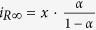 (see Eq. (5)) through implicit UCS revaluation learning, and, hence, through the successive updatings of the reactive response by error signal computations (see Eqs (6 and 7)). Furthermore, if a placebo administration trial follows, then the implicit placebo response, which is equal to the asymptotic value *i*_*R*∞_, is experienced, moreover, the experienced outcome is updated and an error signal is computed, leading to a decrease of the reactive (placebo) response, which vanishes over successive placebo trials. However, if the error signal computation is blocked or “degraded” during placebo (or lower active dose) administration, the experienced outcome is updated; conversely the reactive response (and hence the implicit placebo response) does not decrease (or decrease by little), since no error signal (or a degraded error signal) updated it. Furthermore, a resistant-to-extinction reactive response can be obtained, since no error signals can be computed over successive trials, because the expected outcome coincides with the reactive response, which, in turn, coincides with the experienced outcome; in other words, a reactive response which does not tend asymptotically to zero over successive placebo trials is obtained. We denote such a response *i*_*R*0_, since it will be elicited by the reactive system at every substance intake trial (as it were originally generated by a fictitious trial *zero*), regardless of whether an active pharmacological effect exists. Indeed, it is easy to demonstrate that, if a resistant-to-extinction reactive response *i*_*R*0_ has been associated to a given substance, the expression for the response to substance intake driven through implicit learning in the generic n-th trial is:


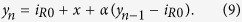


(It has been shown^14^ that different resistant-to-extinction emotional reactive responses are natively coded in the mammalian brain from birth, and these are associated with phylogenetic (i.e., prepared biological and evolutionary fear relevant) stimuli representations^73^,^74^, or with important UCS representations, such as food). The resistant-to-extinction property of a reactive response is the key element for increasing unlimitedly an implicit response; indeed, repeating the cycle of trials (i.e., the above described protocol) adopted for obtaining *i*_*R*0_, will lead to the increase of the reactive response, through an accumulation principle (see Methods for the computational details). Hence, through multiple cycles of active pharmacological drug administration followed by inerte (or a lower active dose) substance administration together with a pharmacological treatment able to degrade the error signal computation (more specifically able to lower the precisions associated with error signals^45^,^46^), it is possible to “force” the CNS to react to such a drug with an increasing self-induced response which mimics the active pharmacological treatment. After some cycles it is expected that the reactive response *i*_*R*_ will be greater than the original active pharmacological response, and the pharmacological effect can be increased indefinitely.

In practice, it is expected that the error signal cannot be completely eliminated in the placebo administration trials, so that multiple placebo administration trials with an “error signal degradation treatment” will be required, and the resulting *i*_*R*0_ will be smaller than *i*_*R*∞_. Nevertheless, the proposed implicit learning model describes the reactive system dynamics at a system (macro) level and, even if it predicts the possibility of obtaining a resistant-to-extinction (i.e., stable) and increasing reactive response, it does not give any indications of how obtaining it, nor which pharmacological targets have to be considered for the degradation of the error signal. For these reasons, a specific model, which can describe the error signal computation at a deeper level (i.e., at the neurons level) is required. We argue such a model could be represented by the so called *predictive coding* model (and its version applied to action learning; *active inference*^44^,^45^).

### Predictive Coding, Active Inference and Error Signal Computation

In predictive coding^44^,^45^ is formalized the notion of the Bayesian brain, in which neural representations in the higher levels of cortical hierarchies generate predictions of representations in lower levels. These *top-down* predictions are compared with representations at the lower level to compute a *prediction error*. The resulting error-signal is passed back up the hierarchy to update higher representations; this recursive exchange of signals lead to the minimization (ideally the suppression) of the prediction error at each and every level to provide a hierarchical explanation for sensory inputs that enter at the lowest (sensory) level. In the Bayesian jargon neuronal activity encodes beliefs or probability distributions over states in the world that causes sensations^45^. In predictive coding the notion of *precision* (or confidence, which represents the inverse of the variance) of the error signals is also formalized, and the mechanism through which the brain has to estimate and encode the precision associated with the prediction errors is explained. The prediction errors are then weighted with their precision before being assimilated at a high hierarchical level. More specifically, active inference accounts posit that the brain deals with *noisy* prediction errors by decreasing the gain on cortical pyramidal neurons that function like precision units to regulate outputs from signal error computations, thereby reducing the influence of these outputs^44^,^75^,^76^. It is important pointing out that the resulting error signal depends on the relative precision of prediction errors at each level of the neural hierarchy. Hence, a neuropharmacological alteration of the precision on different levels in the cortical hierarchy could lead to the modulation of the error signal computation. Furthermore, it is supposed^77^,^78^ that such an alteration may naturally occur in some pathologies, such as in Parkinson’s disease, where the depletion of dopamine (which is supposed to encode the precision of prediction errors by altering their synaptic gain, as also other neurotransmitters do^78^) at different levels, would alter the balance of precision at higher (sensorimotor) relative to lower (primary sensory) levels in the cortical hierarchy; moreover, the symptoms vary according to the site (hierarchical level) of changes in precision^77^.

### Neuronal Populations within the Reactive System

Which are the neuronal populations belonging to the reactive system? In other words, for which CNS response components does the reactive mimicking property hold? If a given neuronal population belongs to the reactive system, then it is possible to obtain a reactive response (i.e., an implicit placebo response) for this neuronal population. Experimental results from the literature evidence that at least the following CNS components could generate a reactive response: 1) emotional system responses^14^ (which include, for instance, the dopaminergic mesolimbic and mesocortical system^79^,^80^, the endocannabinoid and opioid system in placebo analgesia^23^,^25^,^26^,^81^,^82^,^83^,^84^, the serotoninergic system, the target neuronal systems of depression, anxiety and addiction^1^); 2) the dopaminergic motor system^21^,^22^; 3) the *humoral immune response system* (in particular the components of the CNS such as the hypothalamic-pituitary-adrenal axis, HPA, or the sympathetic nervous system, SNS^1^,^85^,^86^,^87^); 4) the endocrine system^1^,^88^. We speculate that the main components of the reactive system are the emotional/motivational and the humoral immune systems, since, from an evolutionary perspective, a given organism has to be able to learn and to automatically react whenever “important” stimuli are perceived, and, implicitly recognize “relevant” substances, such as food, in order to approach or avoid them or to quickly and automatically react (for instance through an automatic trigger of a first immune response). More specifically, a stimulus is “important or relevant” for an organism if it previously elicited a CNS response belonging to the reactive system, such as a painful stimuli (which are related to emotional components), a rewarding substance (such as food), or primary immune responses.

## Discussion

We propose a model describing response to substance intake based on implicit UCS revaluation learning (which describes the automatic evaluation and revaluation of an unconditioned stimulus, that represents the given substance intake). Such a model formalizes the key properties of drug administration response based on well established empirical evidence. The theoretical conceptualization leads to a theorem which proves the necessity of the encoding of two fundamental quantities: the reactive response and the expected (or predicted) outcome. We have derived a model that meets this requirement and is able to predict the response to drug administration over successive trials. The placebo response is mathematically formalized as a reactive response, which represents an implicit and inevitable component of the overall response to substance intake; moreover its effect becomes naturally evident when an organism responds to a substance administration whose active (pharmacological) effect has been eliminated.

Modeling the dynamics of the reactive system, from which the placebo response originates, permits also the definition of quantities (e.g., the reactive efficiency, *α*) and of operational measures which could lead to the maximization and the stabilization of the reactive response (*i*_*R*_) for a given genetic makeup, even when the pharmacological active effect (*x*) is kept constant. Furthermore, the model shows that it should be possible to create a resistant-to-extinction reactive response blocking (or degrading) the error signal computation in specific trials. More specifically, after some trials in which the active drug is administered in order to increase the implicit reactive response, successive trials have to follow, in which placebo administration is given together with a pharmacological treatment able to degrade the precisions of the error signal (and hence the overall error signal computation). The targets of such a pharmacological treatment have to be specific neurotransmitters on precise level of the neural hierarchy, depending on the target component of the original active drug administration; moreover, predictive coding and active inference model provides the theoretical and computational basis for the assessment of such targets.

In this manuscript, the theoretical feasibility study of a strategy for the unlimited increase of a given drug effectiveness has been developed. Further computational and experimental studies are needed in order to explore the mentioned strategy.

We also argue that the generality of our model permits the modeling of a broad range of phenomena and pathologies which involve the interaction between an organism and a substance intake, such as, drug addictions and eating disorders, and for the development of novel strategies for decreasing pathological resistant-to-extinction reactive responses, such as in phobias or post-traumatic stress disorders.

## Methods

In the following sections the equations describing the reactive system dynamics (and, hence, the implicit placebo response dynamics) are derived. It is assumed that no cognitive information processes occur (in other words, verbal suggestions, beliefs and expectations on clinical benefit are avoided); however, drug administration, or substance intake, has to be “overt”, so that the considered individual is aware that the same substance is administered over successive trials (but he/she does not know which is the pharmacological target of such a substance). Furthermore, to ease the computations, it is assumed that the expected (predicted) response, in a given k-th trial, coincides with the last experienced outcome (i.e., *y*_*expected*,*k*_ = *y*_*k*−1_).

### Implicit Response Acquisition to a Substance Intake

In this section the responses elicited during successive trials in which an active drug is administered are derived.

In Table 1 the variables of interest are mathematically described through successive trials. A single CNS component (i.e., a specific target neural population) is considered for the computations, provided that such a component belongs also to the *reactive system*.

The response to drug intake in the n-th trial can be written as:


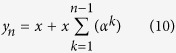


Considering the reactive system stability property, it follows that the term *α* has to be less than one in magnitude (i.e. 0 < |*α*| < 1). Hence the summation term in Eq. (10) converges to the value *α*/(1 − *α*), for the property of the geometric series, when the number of trials tends to infinity. Thus, the asymptotic response due to the active drug administration can be written as:


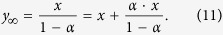


The Eq. (10) can also be expressed as:


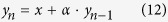


which shows the recursive nature of the response acquisition process over successive trials.

### Reactive or Implicit Placebo Response Dynamics

In this section the response elicited during successive trials where a placebo is administered (i.e. the active pharmacological component is absent) is derived.

In Table 2 the variables of interest are mathematically described over successive trials. A single generic component is considered in the computations, moreover, it is assumed that the asymptotic response has been reached over the previous active drug administration trials (see Eq. (5)).

The response to placebo administration in the n-th trial can be written as:


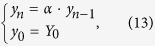


where, provided that the asymptote had been reached during the previous active pharmacological trials, *Y*_0_ = *x* · *α*/(1 − *α*).

**Theorem**: *It is necessary that two distinct quantities are encoded within the CNS for the stability of the reactive system, these are the reactive response and the predicted* (*expected*) *outcome*.

***Proof by contradiction*** (***reductio ad absurdum***)

*Hypothesis* 1: The reactive response associated with a given substance intake coincides with the expected (predicted) outcome, and the expected or predicted outcome converges to the experienced outcome.

*Hypotheses* 2: The properties integration, reactive mimicking and reactive system stability hold.

*Hypotheses* 3: Cognitive information processing are avoided (in other words, no beliefs, expectations for clinical benefits, verbal suggestions and so on, are involved).

Hypothesis 1 asserts that a unique reactive signal predicting the drug administration outcome exists, and that this signal coincides with the reactive response elicited when the drug is administered; furthermore, the predicting signal converges (by learning) to the actual experienced elicitation. The last assumption has been formulated to include a more general scenario than that considered in our initial assumptions, in which the expected outcome coincides with the last experienced outcome. From a mathematical viewpoint, the expected outcome can be computed using any supervised learning method (or, alternatively, TD methods^42^) in which the predicted outcome is evaluated on the basis of the past *m* predictions (i.e., of the predicted outcomes in the last *m* trials) and of the actual outcome, minimizing the error between the prediction and the experienced outcome. Otherwise it can be assumed that the predicted outcome coincides with the last experienced outcome.

1. Let the UCS be the drug administration.

2. Drug administration to a given subject takes place on successive trials, so that it exerts an active elicitation (i.e., it elicits the active response *x*). During the first trial the response is exclusively due to the active pharmacological component, that is *y*_1_ = *x*. After the first trial (for instance, during the drug administration in the second trial), the predicted (reactive) response, called *y*_*predicted*,1_, is computed.

3. In the second trial, after the drug administration, the predicted outcome (*y*_*predicted*,1_) adds up to the successive UCS active elicitation, so that the outcome can be expressed as *y*_2_ = *y*_*predicted*,1_ + *x*. Furthermore, since *y*_*predicted*,1_ does not coincide with the actual experienced outcome, the new prediction *y*_*predicted*,2_ is computed after the second trial; since the error signal is positive it follows that *y*_*predicted*,2_ > *y*_*predicted*,1_

4. In the third trial the experienced outcome can be written as *y*_3_ = *y*_*predicted*,2_ + *x*; since *y*_3_ > *y*_2_ ≥ *y*_*predicted*,2_ a new value for the predicted response is computed, called *y*_*predicted*,3_, such that *y*_*predicted*,3_ > *y*_*predicted*,2_

5. In the *n*-th trial the outcome can be expressed as *y*_*n*_ = *y*_*predicted*,*n*−1_ + *x*; it is easy to prove that *y*_*n*_ > *y*_*n*−1_ ≥ *y*_*predicted*,*n*−1_. Moreover, if the number of trials tends to infinity, the outcome grows indefinitely (i.e., 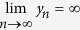).

6. The last statement is absurd, as it contradicts Hypothesis 2, in particular it contradicts the stability system principle.

### Derivation of the continuous time scale model

**Hypothesis** (HC1): the expected response can be computed as a weighted average of the last trials outcomes^89^. This hypothesis is motivated by the consideration that the expected/predicted response can be shaped considering the last previous outcomes and not only the last one (as adopted in the simplified discrete model). This line of reasoning is supported by experimental results^90^ which show that dopamine neurons *encode the difference between the current reward and an exponentially weighted average of previous rewards*.

Under the above mentioned hypothesis, the predicted response, denoted 〈*y*_*n*−1_〉 (since it represents the filtering function of the last responses until that occurred in the n-1 trial), can be expressed as:


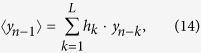


where *L* represents the number of the responses involved in the filtering process, and *h*_*k*_ represents the generic *k*-th filter coefficient (or weight). Note that Eq. (14) expresses a general moving filtering function and not only an exponential one. It can be shown that a moving exponentially averaged filter (in discrete time) represents a *low pass filter* (and also that its continuous-time counterpart is an R-C first order type filter; ref. 91). Substituting Eq. (14) in Eq. (12) yields:


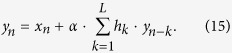


Furthermore, considering that the error signal is now computed as:


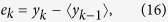


Eq. (15) can also be written as (see also Eqs (2 and 7)):


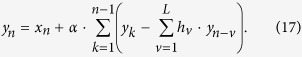


**Adding Hypothesis** (HC2): the pharmacological active stimulation presents a recurrent pattern over successive trials (in other words the pharmacodynamics within a given trial is considered), and such a pattern is periodic with period equals to *N* (so that *x*_*n*_ = *x*_*n*−*N*_).

If the brain recognizes recurrent patterns of stimulation over time (i.e. a typical stimulation intensity pattern), such an information will be exploited for a more precise inference of the probable outcome. This line of reasoning is supported by experimental results which show that neurons encode precise timings between stimuli^41^; for instance, dopamine neurons learn, after few observations, that after a prescribed time from the onset of a cue a given quantity of reward will be delivered.

Learning statistical regularities and patterns represent a type of CC learning, since the variable “time” and “temporal relations” can be considered as contextual cues^92^. From a modeling perspective it is important to note that if the brain “is sure” about the fact that a specific pattern of stimulation is occurring (denoted *y*_*P*(1..*N*)_ where the interval (1..*N*) represents the entire range of trials/time instants the pattern comprises), then the predicted response at the generic *n*-th trial/time instant is represented by the corresponding intensity within the pattern (i.e. *y*_*Pn*_). Conversely, if the brain does not recognize any pattern, no adding inference can be performed and the predicted response is computed as in Eq. (14). Nevertheless, intermediate situations between the above mentioned extremes can occur; more precisely during pattern learning, or whenever the recognized probability of having a given pattern is not equal to one, the expected response has to be computed as a combination of the actual UCS revaluation contribution (Eq. (14)) and of the response expected by the pattern. Hence, the predicted (expected) response can be expressed as:


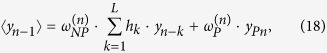


where 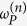 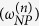 represent the weight (or *belief confidence*) related to the occurrence (no-occurrence) of the given pattern at the *n*-th trial (and they are comprises between 0 and 1). Furthermore, 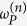 can be seen as the *synaptic strength* associated to the pharmacodynamical curve, which is learned over successive trials, through a *stochastic Hebbian plasticity rule*^14^,^93^,^94^,^95^. Hence, the weights related to the confidence level about the occurrence of the pattern can be computed as^14^:


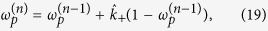


every time the given pattern is recognized (i.e. at every active pharmacological administration), and:


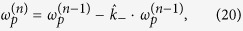


if the pattern does not occur (i.e. whenever a placebo is given so that the previously learned pattern is not observed); and, in both cases:


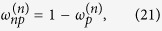


where, the terms 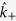 and 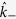 represent the synaptic potentiation and depression rate respectively.

Eq. (18) shows that the predicted response comprises two contributions: a) the UCS revaluation component, which represents the *bottom-up contribution*, since it is determined by the actual implicit UCS revaluation (or its gradient over time) and is exploited from higher level neuronal networks to form more complex hierarchical patterns; 2) the contribution due to previous *inference* (e.g. classical conditioning) learning, which represents the *top-down contribution*, since it is encoded by higher level hierarchical neural structures and exploited to compute the reactive response. Finally, the discrete model with hypothesis HC1-HC2 can be expressed as follows:


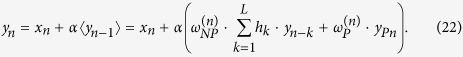


In order to derive the continuous model under the hypothesis of a given pharmacodynamical pattern, denoted *x*(*t*) with time duration equals to *T*_0_ (such that *x*(*t*) = *x*(*t* − *T*_0_), since the pattern can be periodicized over successive drug administration trials), the following procedure is applied to the discrete model (Eq. (22)):

a) it is assumed that the discrete model can be obtained sampling the continuous time counterpart of it with a sampling time *T*, which represents the *sensory time discrimination threshold*^96^. In other words it represents the smallest temporal interval for which the CNS neurons can discriminate between two distinct consecutive stimulations, hence, this parameter is taken as the sampling time.

b) The *transfer function* of the discrete recursive difference system in the Z-domain^91^ is computed; c) the transfer function of the continuous dynamic system is obtained in the *S*-Laplace domain substituting the Z variable of the discrete transfer function with the following equation:


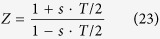


where the variable *s* represents the Laplace variable (this substitution represents the inverse operation of the so called *bilinear transform*^91^); d) the differential equation in the time domain (or the continuous time state space representation) is obtained with the inverse Laplace Transform of the equation in the Laplace domain obtained in the previous stage c).

The model transfer function can be expressed as follows:


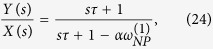


In the first administration trial, and as:


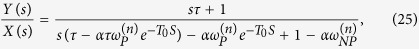


for successive drug administrations, where, *s* represents the Laplace variable; *τ* represents the time constant of the low pass filtering process (which is the equivalent counterpart of the exponential weighted average of the discrete model) which is related to the specific target population (i.e. its value can change depending on the neurotransmitters, neuromodulators and hormones involved in the stimulation) and it can be inferred experimentally monitoring the neuronal target population over time. If the differential equation (or its equivalent state space model) is needed, the term exp(−*T*_0_*S*) in Eq. (25) has to be approximated by a series expansion. Moreover, numerical simulations can be performed adopting the discrete model with a sampling time smaller than τ/20, followed by a numerical interpolation.

### Increasing a Reactive Response through Resistant-To-Extinction Increments

Provided that a resistant-to-extinction reactive response, denoted *i*_*R*0_, has been previously obtained (see Section “Obtaining Increasing and Resistant-to-Extinction Reactive Response”), it is easy to show that the drug intake response is expressed as:


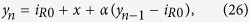


where the term *i*_*R*0_ represents the inextinguishable reactive response obtained during the first protocol cycle. The generic response at the n-th trial can also be expressed as:


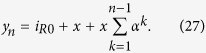


Eqs (26 and 27) can be obtained from the computations shown in Table 3.

## Additional Information

**How to cite this article**: Puviani, L. and Rama, S. Placebo Response is Driven by UCS Revaluation: Evidence, Neurophysiological Consequences and a Quantitative Model. *Sci. Rep.* **6**, 28991; doi: 10.1038/srep28991 (2016).

## Figures and Tables

**Table 1:** In this Table the computations leading to the automatic central nervous system (CNS) response to a drug intake over successive trials are derived.

Trial number	Active pharmacological response (*x*_*k*_)	Reactive response (*i*_*R*,*k*_ = *i*_*R*,*k*−1_ + *α* · *e*_*k*−1_)	CNS response (*y*_*k*_ = *x*_*k*_ + *i*_*R*,*k*_)	Error signal (*e*_*k*_ = *y*_*k*_ − *y*_*k*−1_)
0	*x*_0_ = 0	*i*_*R*0_ = 0	*y*_0_ = 0	*e*_0_ = 0
1	*x*_1_ = *x*	*i*_*R*,1_ = 0	*y*_1_ = *x*	*e*_1_ = *x*
2	*x*_2_ = *x*	*i*_*R*,2_ = *α* · *x*	*y*_2_ = *x* + *α* · *x*	*e*_2_ = *α* · *x*
3	*x*_3_ = *x*	*i*_*R*,3_ = *α* · *x* + *α*^2^ · *x*	*y*_3_ = *x* + *α* · *x* + *α*^2^ · *x*	*e*_3_ = *α*^2^ · *x*
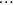	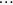	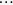	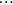	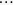
n	*x*_*n*_ = *x*	*i*_*R*,*n*_ = *αx* + *α*^2^*x* + … + *α*^*n*−1^*x*	*y*_*n*_ = *x* + *α* · *x* + *α*^2^*x* + … + *α*^*n*−1^*x*	*e*_*n*_ = *α*^*n*−1^*x*

It is assumed that the active pharmacological effect (*x*) is constant over successive trials.

**Table 2:** In this Table the computations leading to the reactive (implicit placebo) response extinction over successive trials are derived.

Trial number	Active pharmacological response (*x*_*k*_)	Reactive response (*i*_*R*,*k*_ = *i*_*R*,*k*−1_ + *α* · *e*_*k*−1_)	CNS response (*y*_*k*_ = *x*_*k*_ + *i*_*R*,*k*_)	Error signal (*e*_*k*_ = *y*_*k*_ − *y*_*k*−1_)
0	*x*_0_ = *x*	*i*_*R*0_ = *x* · *α*/(1 − *α*)	*y*_0_ = *x*/(1 − *α*)	*e*_0_ = 0
1	*x*_1_ = 0	*i*_*R*,1_ = *x* · *α*/(1 − *α*)	*y*_1_ = *x* · *α*/(1 − *α*)	*e*_1_ = −*x*
2	*x*_2_ = 0	*i*_*R*,2_ = *x* · *α*/(1 − *α*) − *α* · *x*	*y*_2_ = *x* · *α*^2^/(1 − *α*)	*e*_2_ = −*α* · *x*
3	*x*_3_ = 0	*i*_*R*,3_ = *x* · *α*^2^/(1 − *α*) − *α*^2^ · *x*	*y*_3_ = *x* · *α*^3^/(1 − *α*)	*e*_3_ = −*α*^2^ · *x*
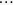	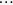	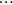	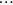	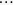
n	*x*_*n*_ = 0	*i*_*R*,*n*_ = *x* · *α*^*n*−1^/(1 − *α*) − *α*^*n*−1^ · *x*	*y*_*n*_ = *x* · *α*^*n*^/(1 − *α*)	*e*_*n*_ = −*α*^*n*−1^*x*

It is assumed that, previously to placebo administration trials, the pharmacological effect was equal to x.

**Table 3:** In this Table the computations leading to the automatic central nervous system (CNS) response to a drug intake over successive trials are derived.

Trial number	Active pharmacological response (*x*_*k*_)	Reactive response (*i*_*R*,*k*_ = *i*_*R*,*k*−1_ + *α* · *e*_*k*−1_)	CNS response (*y*_*k*_ = *x*_*k*_ + *i*_*R*,*k*_)	Error signal (*e*_*k*_ = *y*_*k*_ − *y*_*k*−1_)
0	*x*_0_ = 0	*i*_*R*,0_ = *i*_*R*0_	*y*_0_ = *i*_*R*0_	*e*_0_ = 0
1	*x*_1_ = *x*	*i*_*R*,1_ = *i*_*R*0_	*y*_1_ = *x* + *i*_*R*0_	*e*_1_ = *x*
2	*x*_2_ = *x*	*i*_*R*,2_ = *i*_*R*0_ + *α* · *x*	*y*_2_ = *x* + *α* · *x* + *i*_*R*0_	*e*_2_ = *α* · *x*
3	*x*_3_ = *x*	*i*_*R*,3_ = *i*_*R*0_ + *α* · *x* + *α*^2^ · *x*	*y*_3_ = *x* + *α* · *x* + *α*^2^ · *x* + *i*_*R*0_	*e*_3_ = *α*^2^ · *x*
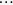	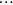	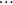	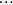	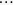
n	*x*_*n*_ = *x*	*i*_*R*,*n*_ = *i*_*R*0_ + *αx* + *α*^2^*x* + … + *α*^*n*−1^*x*	*y*_*n*_ = *x* + *α* · *x* + *α*^2^*x* + … + *α*^*n*−1^*x* + *i*_*R*0_	*e*_*n*_ = *α*^*n*−1^*x*

It is assumed that the active pharmacological effect is constant and equal to *x*, furthermore, a resistant-to-extinction reactive response (*i*_*R*0_) associated to the given substance intake has been previously obtained, through the strategy described in the previous sections.
